# Glutathione Fine-Tunes the Innate Immune Response toward Antiviral Pathways in a Macrophage Cell Line Independently of Its Antioxidant Properties

**DOI:** 10.3389/fimmu.2017.01239

**Published:** 2017-09-29

**Authors:** Marina Diotallevi, Paola Checconi, Anna Teresa Palamara, Ignacio Celestino, Lucia Coppo, Arne Holmgren, Kahina Abbas, Fabienne Peyrot, Manuela Mengozzi, Pietro Ghezzi

**Affiliations:** ^1^Brighton and Sussex Medical School, Brighton, United Kingdom; ^2^Department of Public Health and Infectious Diseases, Sapienza University of Rome, Laboratory Affiliated to Istituto Pasteur Italia—Fondazione Cenci Bolognetti, Rome, Italy; ^3^IRCCS, San Raffaele Pisana, Telematic University, Rome, Italy; ^4^Department of Medical Biochemistry and Biophysics, Karolinska Institutet, Stockholm, Sweden; ^5^LCBPT, UMR 8601 CNRS—Paris Descartes University, Sorbonne Paris Cité, Paris, France; ^6^ESPE of Paris, Paris Sorbonne University, Paris, France

**Keywords:** inflammation, innate immunity, TLR4, macrophages, glutathione, redox regulation, antiviral immunity, influenza

## Abstract

Glutathione (GSH), a major cellular antioxidant, is considered an inhibitor of the inflammatory response involving reactive oxygen species (ROS). However, evidence is largely based on experiments with exogenously added antioxidants/reducing agents or pro-oxidants. We show that depleting macrophages of 99% of GSH does not exacerbate the inflammatory gene expression profile in the RAW264 macrophage cell line or increase expression of inflammatory cytokines in response to the toll-like receptor 4 (TLR4) agonist lipopolysaccharide (LPS); only two small patterns of LPS-induced genes were sensitive to GSH depletion. One group, mapping to innate immunity and antiviral responses (Oas2, Oas3, Mx2, Irf7, Irf9, STAT1, il1b), required GSH for optimal induction. Consequently, GSH depletion prevented the LPS-induced activation of antiviral response and its inhibition of influenza virus infection. LPS induction of a second group of genes (Prdx1, Srxn1, Hmox1, GSH synthase, cysteine transporters), mapping to nrf2 and the oxidative stress response, was increased by GSH depletion. We conclude that the main function of endogenous GSH is not to limit inflammation but to fine-tune the innate immune response to infection.

## Introduction

Several studies have concluded that oxidative stress, due to increased production of reactive oxygen species (ROS), for instance, because of infection, can trigger inflammation, although the concept has been considered an oversimplification (1). This hypothesis is largely based on studies showing that exogenously added ROS induce inflammatory cytokines, while addition of antioxidants, including the main thiol antioxidant, glutathione (GSH), inhibits it. This led to the view of ROS as pro-inflammatory mediators and GSH as an anti-inflammatory mediator (2, 3). However, although animal studies have shown a protective effect of GSH or its precursors in animal models of inflammatory diseases, such as sepsis or acute respiratory distress syndrome (4, 5), this was not confirmed in clinical trials (6). Furthermore, physiological concentrations of ROS, which may not result in oxidative damage, as well as changes in the redox state of cellular thiols, are implicated in biochemical signaling, and administration of antioxidants could disrupt all these redox-dependent signaling mechanisms (7–9).

While in the context of oxidative stress GSH acts as an antioxidant ROS scavenger, in the context of redox regulation the couple GSH/GSSG (oxidized GSH) acts as a signaling molecule that regulates protein function *via* thiol–disulfide exchange reactions including protein glutathionylation (10).

To investigate the role of endogenous GSH in inflammation, whether it acts as an antioxidant or a signaling molecule, we used the mouse macrophage RAW cell line stimulated with lipopolysaccharide (LPS) with and without pretreatment with the GSH synthesis inhibitor buthionine sulfoximine (BSO). GSH/GSSG levels were measured and LPS-stimulated ROS production was quantified by electron paramagnetic resonance (EPR). BSO is an inhibitor of GSH synthase that has been used to deplete GSH *in vitro*, including in macrophages (11, 12), and is more specific than GSH-depleting agents such as diethylmaleate that can activate nrf2 directly due to its electrophilic properties (13).

We analyzed the gene expression profile and identified patterns of LPS-induced genes that were inhibited by endogenous GSH or that, on the contrary, required GSH for their induction. The results indicate that, contrary to the initial hypothesis, inflammatory genes are not affected by the lack of endogenous GSH. Instead, a small pattern of genes mapping to innate immunity and antiviral activity required GSH for their induction.

## Materials and Methods

### RAW264 Cells, GSH Depletion, and Treatment

RAW264 cells were cultured in Roswell Park Memorial Institute medium 1640 with 2 mM l-glutamine (Sigma), 100 U/ml penicillin, 100 µg/ml streptomycin sulfate (Invitrogen/Life Technologies), and 10% heat-inactivated FCS (Sigma)-omplete medium. Cells were plated at the density of 10^6^/well in 6-well plates. For GSH depletion, BSO was added at the final concentration of 120 µM. After 24 h, control or GSH-depleted cells were stimulated with 10 ng/ml LPS and incubated for the times indicated. When indicated, cells were exposed to menadione sodium bisulfite or *N*-acetyl-l-cysteine (NAC; Sigma) as indicated in the text. Both chemicals were dissolved in phosphate-buffered saline (PBS); the pH value of NAC was adjusted to 7.4 using NaOH.

### GSH Quantification

Glutathione and GSSG were measured in lysates from cells in 6-well plates as previously described (14). Protein content was measured using the Bradford reagent (15) and GSH/GSSG levels expressed as nmol/mg protein.

### ROS Quantification by Spin-Trapping and EPR Spectroscopy

Cells (5 × 10^6^/0.1 ml) were stimulated for 2 h with LPS, then incubated with 50 mM 5-tert-butoxycarbonyl-5-methyl-1-pyrroline-N-oxide (BMPO), synthetized as described previously (16), in PBS containing 1 mM diethylenetriaminepentaacetic acid. EPR analysis was performed as described previously (17, 18). EPR intensity of the BMPO adduct in each sample was derived from the sum of 40 scans and expressed as arbitrary units.

### Cell Viability Assay

Cells were seeded in 96-well plates at 25,000/well in complete medium. After overnight culture, BSO at the final concentration of 120 µM was added and the cells were incubated for further 24 h before treatment with or without 10 ng/ml of LPS. After 6 h, cell viability was measured with CellTiter-Blue^®^, following the instructions of the manufacturer (Promega).

### RNA Isolation

Cells were washed with PBS without Ca^2+^ and Mg^2+^ (Sigma) and each sample (1 × 10^6^ cells) was lyzed with 1 ml QIAzol (QIAGEN). Total RNA was extracted by using the miRNeasy system and protocol (QIAGEN). RNA purity and integrity were determined using a NanoDrop ND-1000 (NanoDrop Technologies) and an Agilent 2100 Bioanalyzer (Agilent Technologies). All samples had a A260/A280 ratio > 1.8 and RNA integrity number (RIN) 10. Experiments were performed in quadruplicate; three random samples for each experimental condition were used for microarray analysis and all the four samples for quantitative polymerase chain reaction (qPCR) validation. In total, 24 arrays were done: 3 controls, 3 LPS, 3 BSO, and 3 BSO + LPS at each time point (2 and 6 h).

### Microarray Hybridization

RNA was amplified, labeled, and hybridized onto Single Color SurePrint G3 Mouse GE 8 × 60K Microarrays (AMADID:046066; Agilent Technologies) at Oxford Gene Technology, Oxford, UK, following the instructions of the manufacturer. Following hybridization, the arrays were scanned to derive the array images. Feature extraction software v10.7.3.1 was used to generate the array data from the images.

### Microarray Data Analysis

Raw data in standard format from the microarray experiment have been deposited in the Gene Expression Omnibus (GEO) database of NCBI^1^ under accession n. GSE79397. Raw data were normalized and analyzed using GeneSpring software (Agilent Technologies). Transcript expression between the experimental groups was compared by Student’s *t*-test done on the log_2_ of the gProcessed signal. Fold change in the expression represents the ratio between the averages of the gProcessed signals of the various groups and is expressed as log_2_.

The initial selection was done using the Student’s *t*-test and was based on a binary comparison (genes significantly different between LPS + BSO and LPS alone were selected). These were then selected further using the one-way analysis of variance (ANOVA) test, followed by a correction for multiple comparisons by controlling the false discovery rate with the two-stage step-up method of Benjamini, Krieger, and Yekutieli, as recommended by the GraphPad Prism software (version 7.0 for Mac OS X).

Hierarchical cluster analysis was performed using Genesis software version 1.8.1 for Windows (19). Functional annotation and biological term enrichment to identify the overrepresented gene ontology biological processes (GO:BP) categories and KEGG pathways was done using DAVID software version 6.8.^2^ DAVID calculates a modified Fisher’s exact *P*-value to demonstrate enrichment. Categories with *P* < 0.05 were considered significantly enriched.

### Microarray Data Validation

Reverse transcription (RT) and qPCR were carried out as reported (20) on total RNA from quadruplicate samples. PCR reaction were run on the MX3000 PCR machine (Stratagene/Agilent), using Taqman^®^ gene expression assays (Applied Biosystems/Life Technologies) and Brilliant III qPCR master mix (Stratagene/Agilent Technologies). Gene expression was quantified using the comparative threshold cycle method, according to Applied Biosystems’ guidelines. Results were normalized to HPRT1 expression (reference gene) and expressed as relative expression (fold change) vs one of the control samples at 2 or 6 h (as indicated), chosen as the calibrator.

### Influenza Infection

After 18-h treatment with 120 µM BSO and subsequent 2-h treatment with 10 ng/ml LPS, RAW cells were infected with Influenza A/Puerto Rico/8/34 H1N1 virus (PR8) at a multiplicity of infection (MOI) of 4. The cells were incubated with the virus for 1 h at 37°C in serum-free medium, washed with PBS, and then medium with 2% FBS was added for 24 h. BSO was present in the medium for the 24-h infection. The cell lysates were run on reducing sodium dodecyl sulfate polyacrylamide gel electrophoresis (12% acrylamide) and analyzed by Western blot with anti-influenza antibody (Merck Millipore AB1074), as described previously (21).

## Results

### Effect of GSH Depletion on the Gene Expression Profile of Control or LPS-Stimulated Macrophages

**Figure 1:**
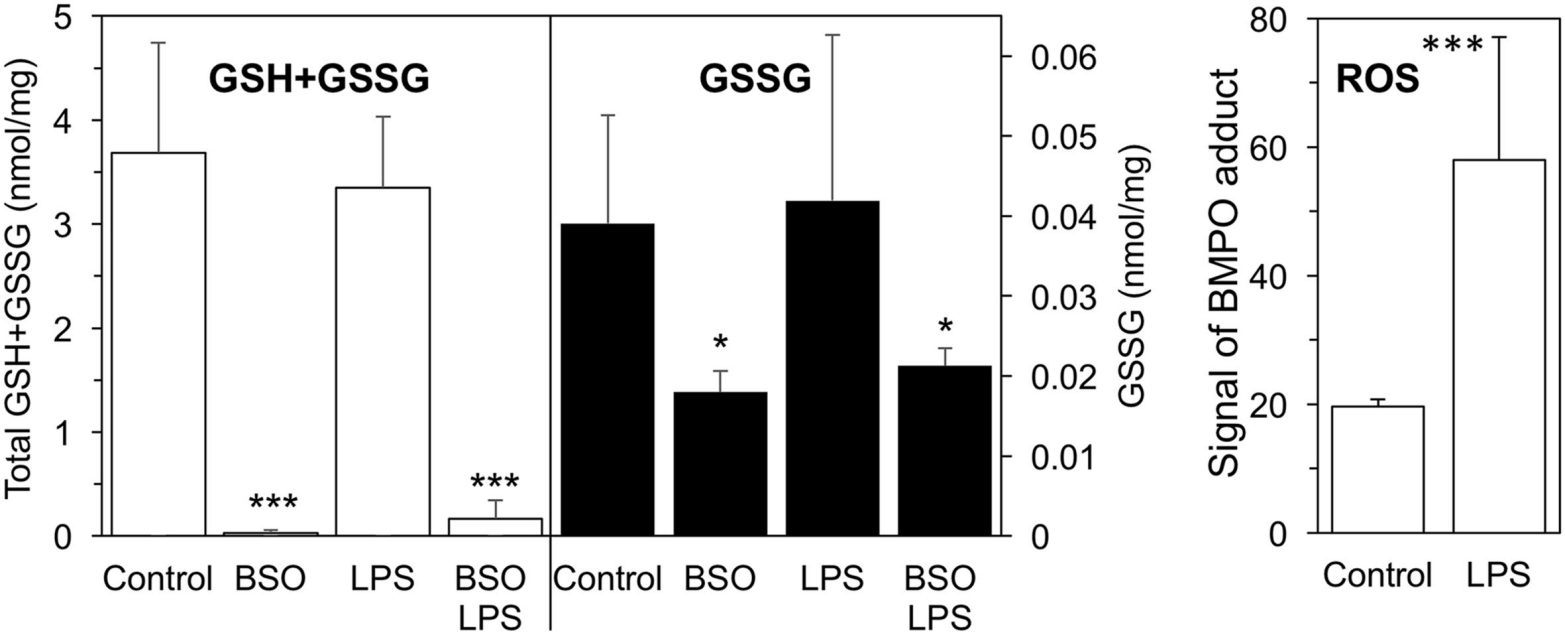
GSH + GSSG levels and ROS production in LPS-treated cells with or without BSO. Cells were pretreated with 120 µM BSO for 24 h, followed by 10 ng/ml LPS for 2 h. Total GSH + GSSG and GSSG levels measured in the cell lysates are the mean ± SD of six biological replicates from three independent experiments and are expressed as nmoles/mg protein. ROS production is expressed as signal of BMPO adduct, mean ± SD of six biological replicates from six independent experiments *P < 0.05 and ***P < 0.001 vs control by two-tailed Student’s *t*-test. BMPO, 5-tert-butoxycarbonyl-5-methyl-1-pyrroline N-oxide; BSO, buthionine sulfoximine; GSH, glutathione; GSSG, glutathione disulfide; LPS, lipopolysaccharide; ROS, reactive oxygen species.

As shown in Figure 1, a 24-h BSO pretreatment decreased glutathione (GSH + GSSG) levels by 99%, and addition of LPS had no effect up to 2 h later. Although LPS induced an oxidative burst in terms of increased ROS production detectable by EPR, this did not affect GSH or GSSG levels significantly. BSO, alone or with LPS, was not toxic to the cells as detected by CellTiter-Blue^®^ Assay (viability, mean ± SD from five biological replicates: control, 100 ± 1%; BSO, 98 ± 2%; BSO + LPS, 95 ± 1%).

**Figure 2:**
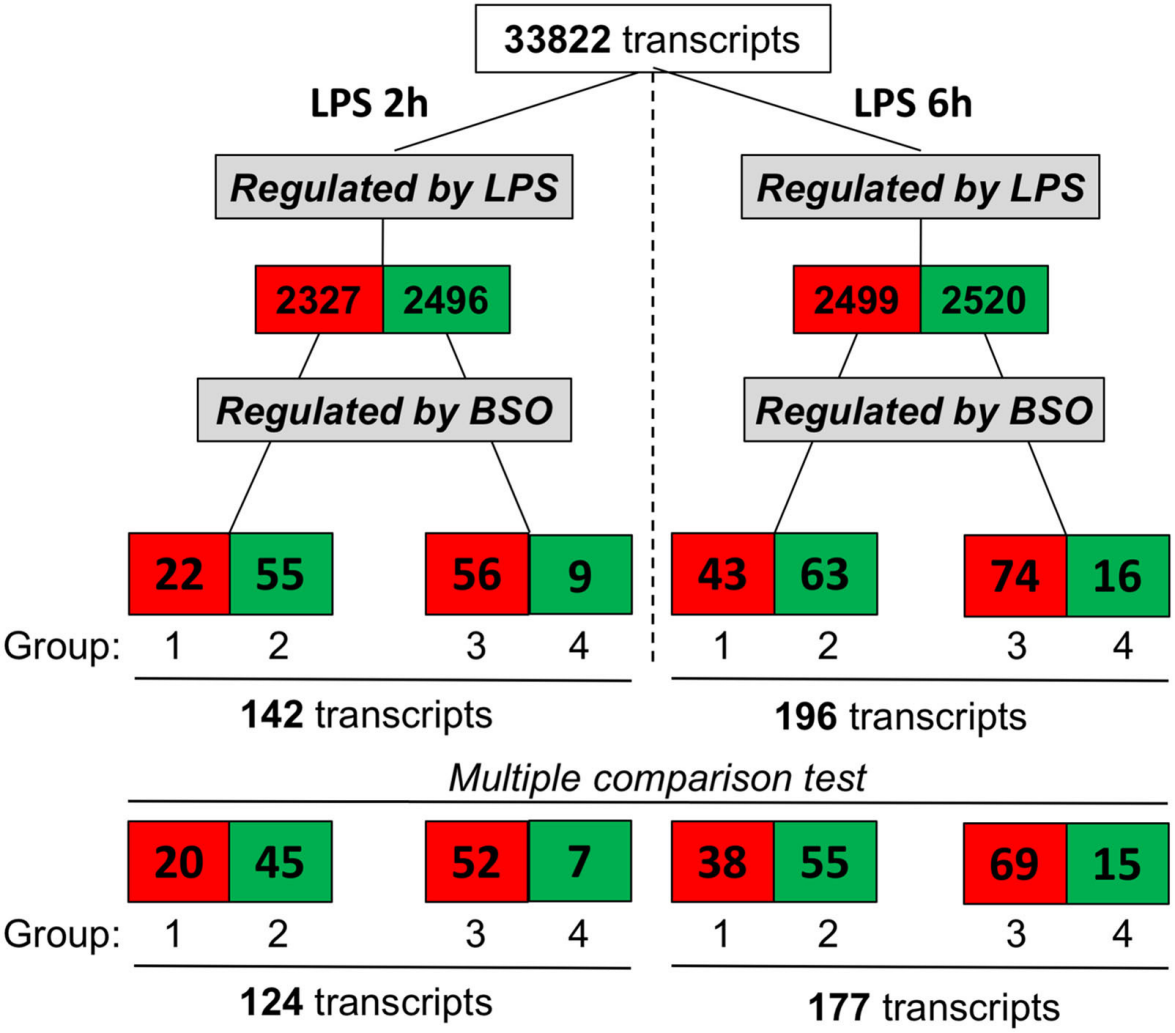
Effect of GSH depletion on LPS-induced changes in gene expression profile. The dataset was filtered to select transcripts that were affected by LPS (LPS vs control, first step), then by BSO (BSO + LPS vs LPS alone, second step). Cutoff for selection was 1.5-fold change and *P* < 0.05, using the Student’s *t*-test. Significantly different expressed genes were further selected by using one-way ANOVA, followed by a correction for multiple comparisons by controlling the false discovery rate with the two-stage step-up method of Benjamini, Krieger, and Yekutieli. The number of transcripts resulting from filtering is indicated and color coded (red, increased; green, decreased). ANOVA, analysis of variance; BSO, buthionine sulfoximine; GSH, glutathione; LPS, lipopolysaccharide.

We exposed cells to BSO and LPS and analyzed the gene expression profile. As outlined in Figure 2, we selected genes whose expression was significantly affected by LPS compared with control cells, using as cutoff a fold change of 1.5 and a significance level of *P* < 0.05; the numbers of upregulated genes are in red, and those downregulated are in green.

Lipopolysaccharide affected about 5,000 transcripts at each time point, with an almost identical number of upregulated and downregulated ones. Of the transcripts affected by LPS, we selected those up- or downregulated by BSO (significantly different by fold change of 1.5 and a significance level of *P* < 0.05 when comparing BSO + LPS vs LPS alone).

At both time points, we could identify four groups of transcripts: (1) upregulated by LPS and increased further by BSO; (2) upregulated by LPS and decreased by BSO; (3) downregulated by LPS and increased by BSO; (4) downregulated by LPS and decreased further by BSO.

While the initial selection was done using the Student’s *t*-test and a binary comparison (BSO + LPS vs LPS alone), because subsequent analysis would also look at the effect of BSO alone, we further selected the genes to be used for subsequent analysis (bottom line in Figure 2) using one-way ANOVA followed by a correction for multiple comparisons, as indicated in Section “Materials and Methods.”

**Figure 3:**
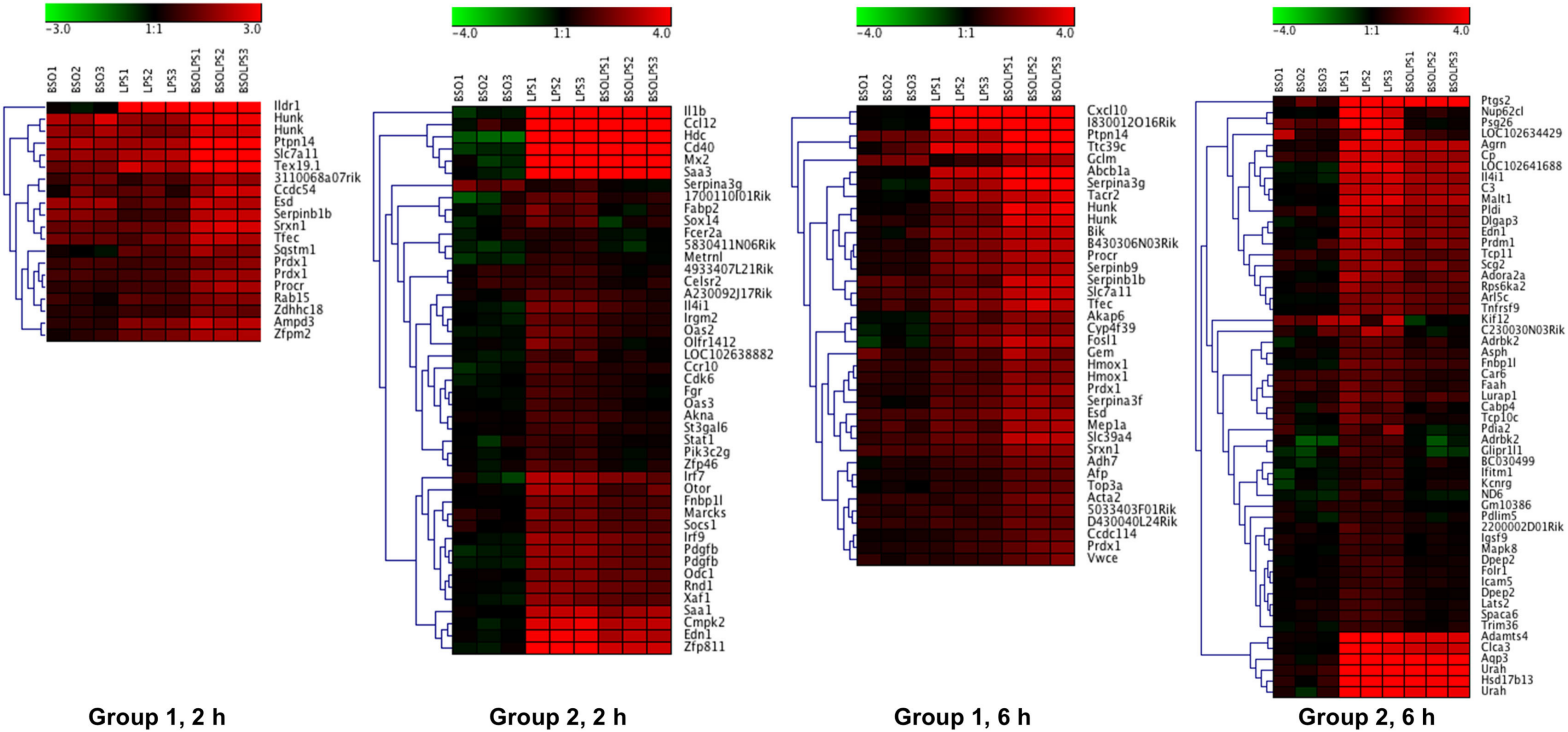
Patterns of gene expression. Cluster heat maps of transcripts in Groups 1 and 2. For each sample (3 BSO, 3 LPS, 3 BSO + LPS), the log_2_ fold change in gene expression vs the mean of three controls is represented. LPS, lipopolysaccharide; BSO, buthionine sulfoximine.

A cluster analysis of the LPS-induced transcripts affected by BSO (Groups 1 and 2) is shown in Figure 3 and it can be seen that a small group of the genes in Group 1 are also increased by BSO alone. On the other hand, BSO alone has no significant effect on genes in Group 2.

The whole list of the 124 + 177 LPS-regulated transcripts affected by BSO at 2 and 6 h is provided in File S1 in Supplementary Material. It can be noted that, for most of the transcripts regulated by LPS, BSO antagonized the effect of LPS on gene expression rather than amplifying it, showing that the effect of BSO was merely additive.

The top 15 transcripts upregulated by LPS that were most affected by BSO in Groups 1 and 2 at the two time points are listed in Table 1.

**Table 1:** LPS-induced transcripts most affected by BSO in Groups 1 and 2.

2 h				6 h			
GeneSymbol	Genbank accession	*P*-value	log_2_FC	GeneSymbol	Genbank accession	*P*-value	log_2_FC
**Most up-regulated by BSO (Group 1)**							

Ptpn14^a^	NM_008976	<0.0001	1.85	Hunk	NM_015755	<0.0001	1.97
Serpinb1b^a^	NM_173052	<0.0001	1.60	Serpinb1b^a^	NM_173052	0.0002	1.85
Ccdc54^a^	NM_027046	0.0100	1.31	Gclm^a^	NM_008129	<0.0001	1.80
**Srxn1**^a^	**NM_029688**	<0.0001	**1.28**	Hunk	NM_015755	<0.0001	1.73
Tex19.1^a^	NM_028602	<0.0001	1.22	Ptpn14^a^	NM_008976	<0.0001	1.52
Hunk^a^	NM_015755	0.0007	1.14	Serpina3g	NM_009251	0.0017	1.42
Esd^a^	NM_016903	0.0002	1.14	Slc39a4^a^	NM_028064	<0.0001	1.40
Procr	NM_011171	<0.0001	1.13	Mep1a^a^	NM_008585	0.0032	1.21
Tfec^a^	NM_031198	0.0046	1.03	Tfec^a^	NM_031198	0.0026	1.14
Slc7a11^a^	NM_011990	<0.0001	1.00	**Prdx1**	**NM_011034**	<0.0001	**1.12**
3110068a07rik^a^	AK039947	0.0033	0.97	Fosl1	**NM_010235**	0.0082	**1.11**
Prdx1^a^	NM_011034	<0.0001	0.97	Gem^a^	**NM_010276**	0.0275	**1.09**
Hunk^a^	NM_015755	0.0008	0.94	**Slc7a11**^a^	**NM_011990**	<0.0001	**1.07**
Zdhhc18	NM_001017968	<0.0001	0.70	Acta2^a^	NM_007392	<0.0001	1.01
Zfpm2	NM_011766	0.0006	0.69	Vwce	NM_027913	0.0009	1.00

**Most down-regulated by BSO (Group 2)**							

Sox14	NM_011440	0.0093	−1.52	Nup62cl	NM_001081668	<0.0001	−4.34
Olfr1412	NM_146277	0.0005	−1.51	Psg26^a^	NM_001029893	<0.0001	−4.21
il1b	**NM_008361**	0.0004	−**1.49**	Kif12^a^	NM_010616	0.0052	−2.56
Fabp2	NM_007980	0.0093	−1.48	LOC102634429	XM_006521612	0.0244	−2.29
**Mx2**	**NM_013606**	0.0329	−**1.39**	C230030N03Rik	AK082264	0.0034	−2.22
Otor	NM_020595	0.0017	−1.29	Pdia2	NM_001081070	0.0068	−1.79
Zfp811	NM_183177	0.0021	−1.13	Adrbk2	AK048763	0.0125	−1.65
Edn1	NM_010104	0.0003	−1.04	Glipr1l1	NM_027018	0.0052	−1.56
Il4i1	NM_010215	0.0003	−1.04	Urah	NM_029821	0.0103	−1.36
**Oas2**	**NM_145227**	<0.0001	−**1.03**	Urah	NM_029821	0.0168	−1.25
5830411N06Rik	AK030813	0.0022	−1.00	Cabp4	NM_144532	0.0129	−1.20
Zfp46	NM_009557	0.0031	−0.99	Tcp10c	NM_001167578	0.0050	−1.18
Irf7	NM_016850	0.0383	−0.92	ND6	AK140300	<0.0001	−1.13
Saa3	NM_011315	0.0015	−0.89	Adora2a	NM_009630	0.0021	−1.07
Pdgfb	NM_011057	0.0002	−0.86	Faah^a^	NM_010173	0.0023	−0.94

*Transcripts were selected for their differential expression between the two groups BSO + LPS vs LPS alone (cutoff was: FC, 1.5; *P* < 0.05 by one-way ANOVA, followed by a correction for multiple comparisons by controlling the false discovery rate with the two-stage step-up method of Benjamini, Krieger, and Yekutieli). Data for both 2 and 6 h are reported. Only the top 15 transcripts most affected by BSO (sorted by FC) are shown*.

*^a^Transcripts that are also significantly affected by BSO alone (BSO vs control, with a cutoff of FC 1.5, *P* < 0.05 as above). The full list can be seen in File S1 in Supplementary Material. Transcripts in bold were selected for PCR validation*.

*ANOVA, analysis of variance; BSO, buthionine sulfoximine: FC, fold change; LPS, lipopolysaccharide*.

As expected, among the transcripts upregulated by LPS and increased further by BSO (Group 1) are several stress defense genes such as peroxiredoxin 1 (Prdx1), sulfiredoxin (Srxn1), heme oxygenase 1 (Hmox1), and genes involved in GSH synthesis including glutamate–cysteine ligase modifier subunit (Gclm) and solute carrier family 7 members 11 (Slc7a11). Of note, these genes were also upregulated by BSO alone.

Interestingly, among the genes upregulated by LPS and decreased by BSO (Group 2), we found genes important in innate immunity and inflammation (il1b, Irf7, Irf9, Mx2, Oas2, Oas3, Ptgs2), as well as the secreted l-phenylalanine oxidase, il4i1. None of these genes were affected by BSO alone.

The list of the top 15 transcripts most affected by BSO among those downregulated by LPS is available as Table S1 in Supplementary Material.

### Functional Categories Differentially Regulated by BSO and LPS

The general functions of the four groups of genes differentially regulated by GSH depletion and LPS were then analyzed using DAVID to identify the enriched GO:BP categories and KEGG pathways (22). For this purpose, we combined the list of differentially expressed genes at 2 and 6 h.

**Figure 4:**
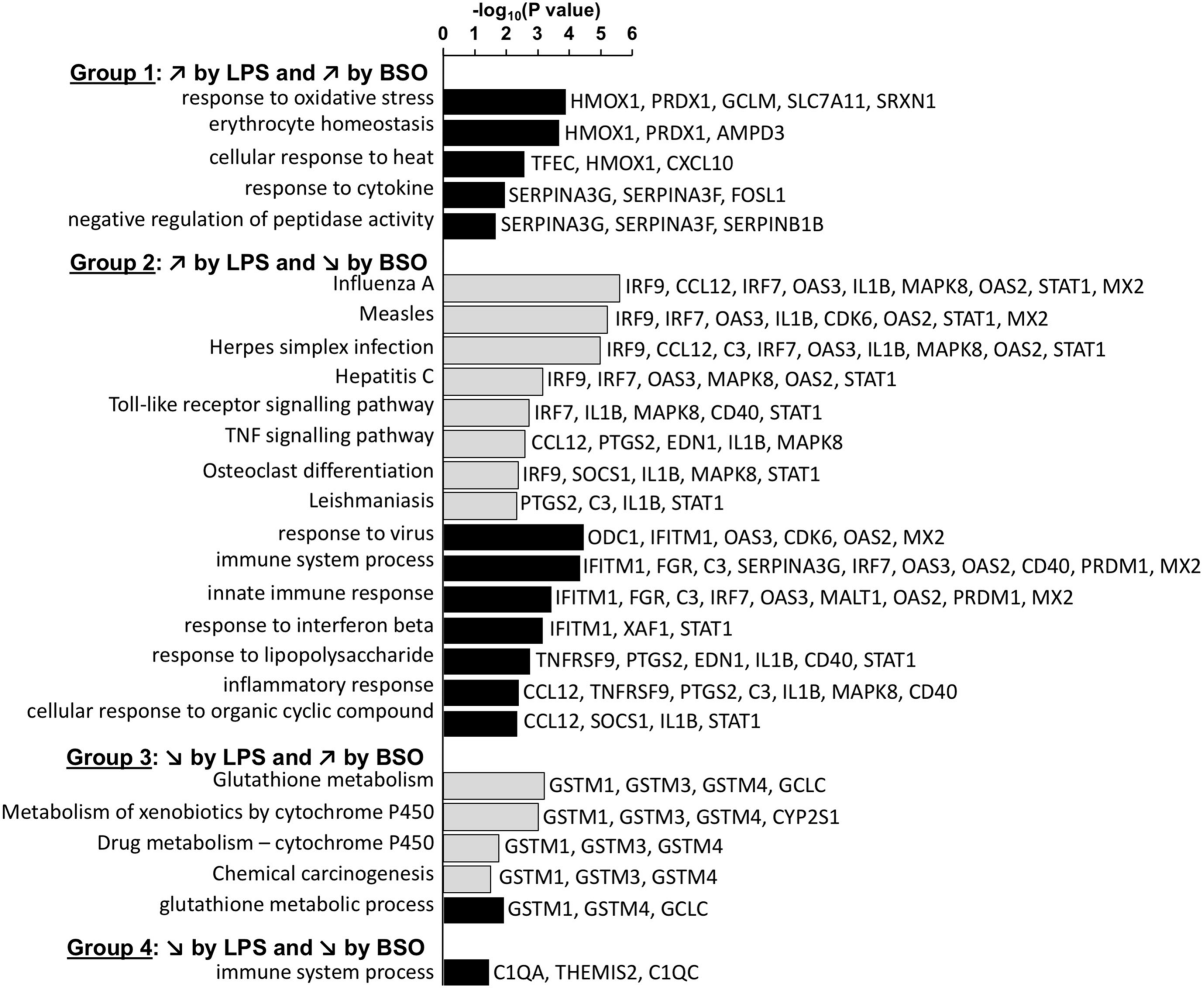
Enriched functional categories in the four groups of genes differentially regulated by LPS and BSO. The lists of genes in the four groups at 2 and 6 h were combined and the overrepresented GO biological process (GO:BP) categories (white bars) and KEGG pathways (gray bars) were obtained by DAVID analysis. All categories identified by DAVID for Groups 1–4 are reported. For Group 2, DAVID returned 51 categories and only the top 15 (ordered by EASE score, a modified Fisher’s exact test) are reported. BSO, buthionine sulfoximine; GO:BP, gene ontology biological process; LPS, lipopolysaccharide.

Figure 4 shows the KEGG and GO:BP categories overrepresented in each of the four groups. Only categories that included three or more genes are shown. The analysis confirms that Group 1 included genes associated with the response to oxidative stress. Group 2 included genes associated with immune response, inflammation, and antiviral host defense such as interferon (IFN) and toll-like receptor (TLR) signaling.

Among the genes whose expression was inhibited by LPS (Groups 3 and 4), only few mapped to some functional category. Group 3 included genes associated with xenobiotics metabolism such as GSH transferases mu 1–4 and cytochrome P450. The only genes that were part of a functional category in Group 4 were C1q components.

### Transcription Factor (TF) Analysis

To identify possible common molecular mechanisms responsible for the differential regulation by BSO of the LPS-induced genes in Groups 1 and 2, we performed an unbiased analysis for the overrepresented TF-binding sites using oPOSSUM software (23).

**Figure 5:**
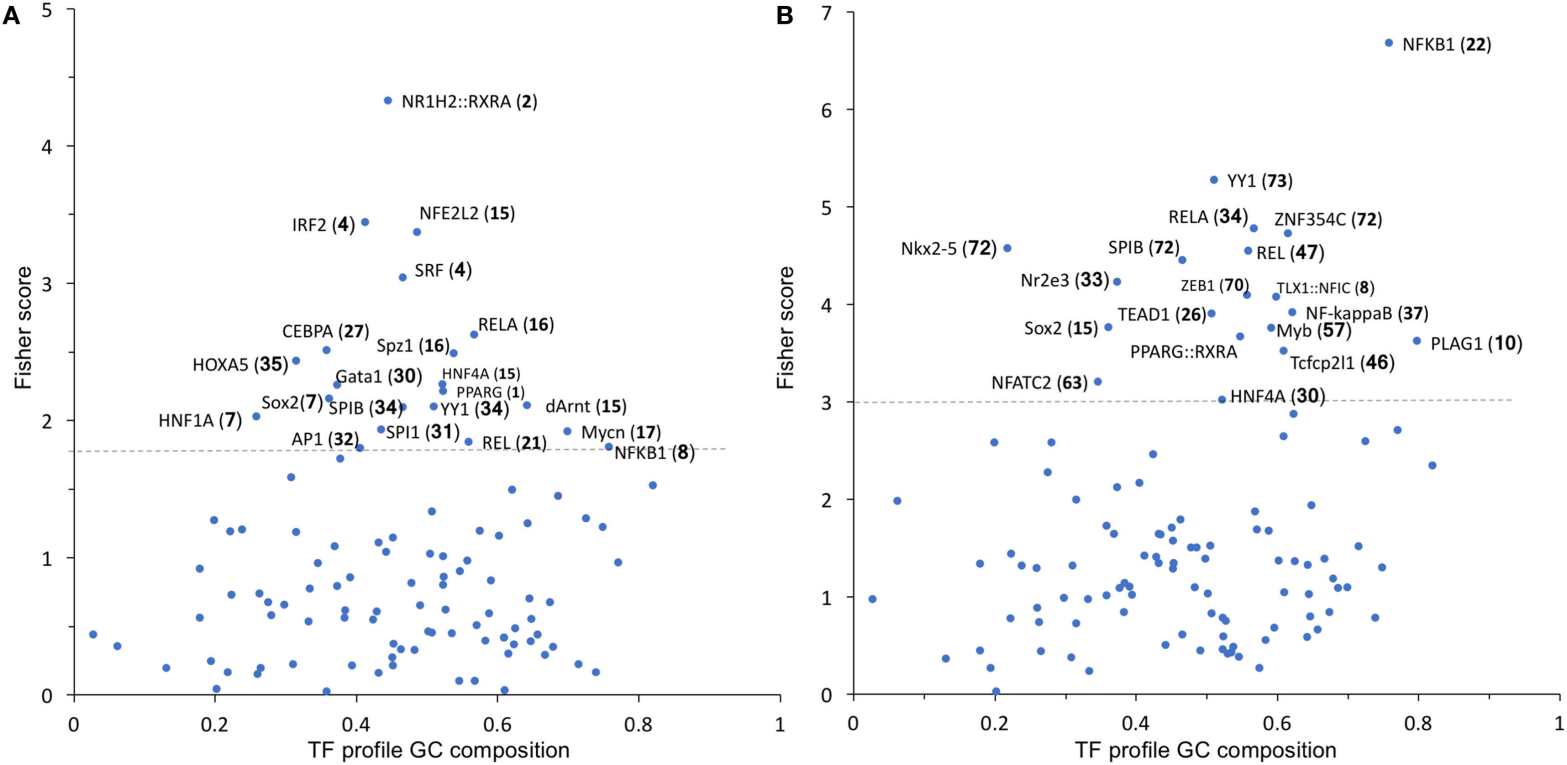
TF binding profile overrepresented in Group 1 **(A)** and Group 2 **(B)** transcripts, showing the Fisher score plotted against GC composition (1 = 100%). The threshold (dashed line) is set to the mean + 1 SD. The number in parentheses indicates the number of transcripts that map to each TF. TF, transcription factor.

In Group 1 (Figure 5A), the TF results in the highest Fisher score and a high number of target genes was NFE2L2 (nrf2), whose main function is the response to oxidative stress, thus confirming the results obtained with DAVID. In Group 2 (Figure 5B), the TF that had the highest score was NF-kB with its various subunits.

We thus manually searched our dataset for the expression of known NF-kB target genes. From the list of 364 genes available at https://www.bu.edu/nf-kb/gene-resources/target-genes/, we could identify, in our dataset and with a cutoff of 1.5-fold change and *P* < 0.05 significance level, 87 NF-kB target genes induced by LPS at 2 h and 107 at 6 h (File S2 in Supplementary Material). However, only 8 out of 87 at 2 h and 4 out of 107 at 6 h were downregulated by BSO. Thus, because only a very small percentage of NF-kB target genes induced by LPS are in Group 2 (downregulated by BSO), we could rule out that BSO acts simply by downregulating NF-kB.

### PCR Validation

**Figure 6:**
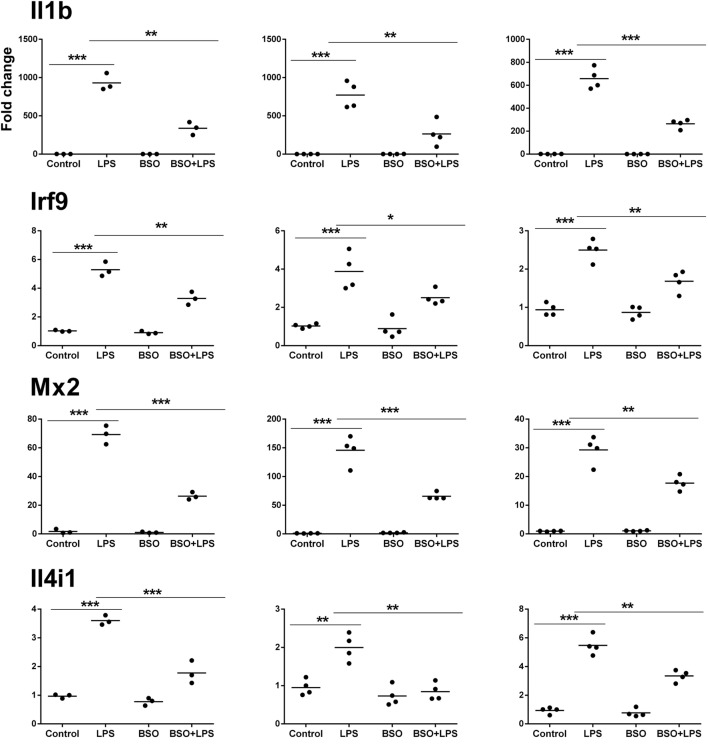

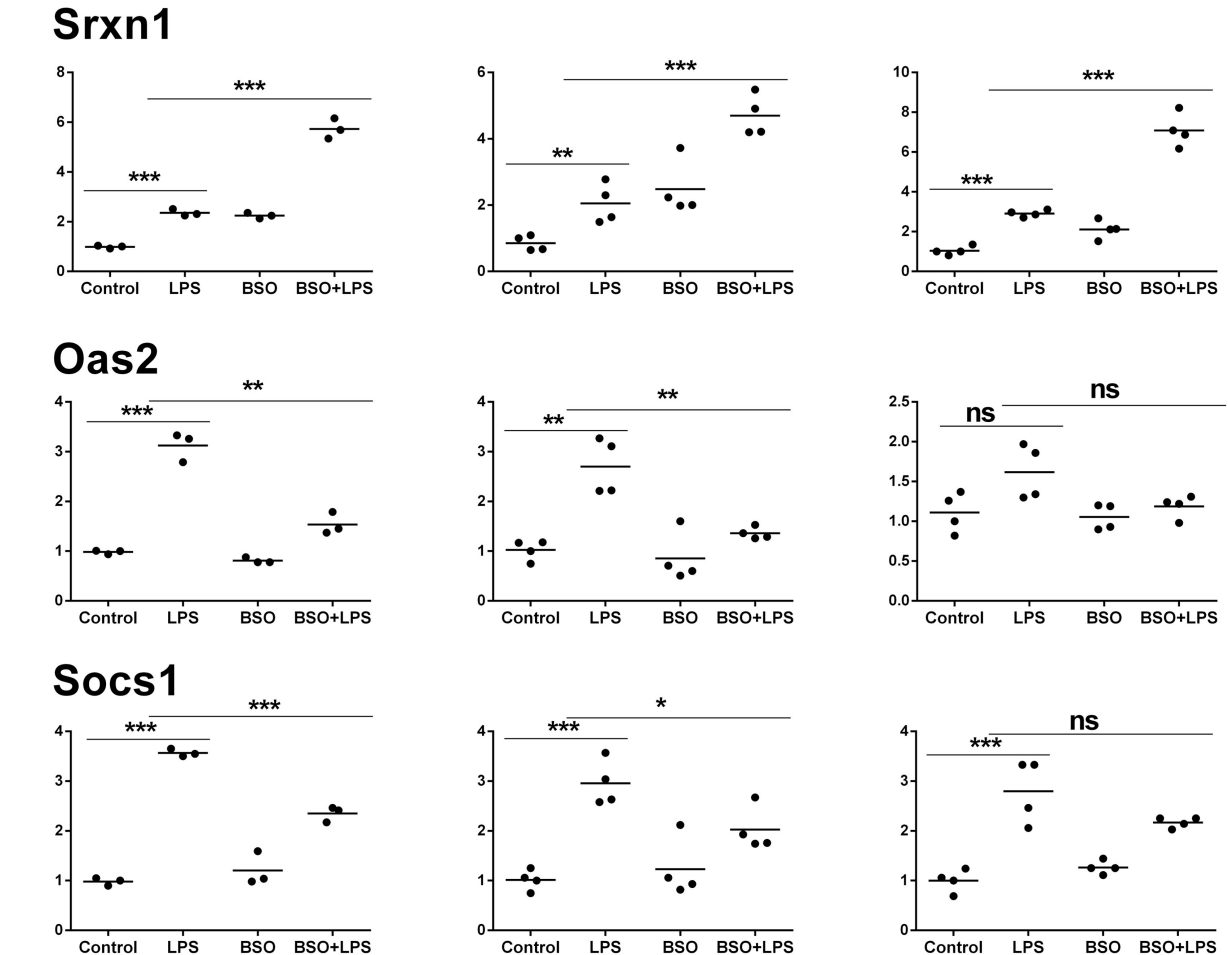
PCR validation of the microarray data at 2 h. Results for seven genes (il1b, Irf9, Mx2, Il4i1, Srxn1, Oas2, Socs1), comparing expression data from microarrays (*N* = 3 biological replicates; left graphs) with results from PCR analysis using all the four replicates of the RNA from the same experiment (*N* = 4 biological replicates; middle graphs) and RNA from an independent experiment (*N* = 4 biological replicates; right graphs). Data are expressed as fold change vs one of the respective control samples. For each experimental group, the mean is also shown. *P < 0.05, **P < 0.01, and ***P < 0.001 by two-tailed Student’s *t*-test. PCR, polymerase chain reaction.

**Figure 7:**
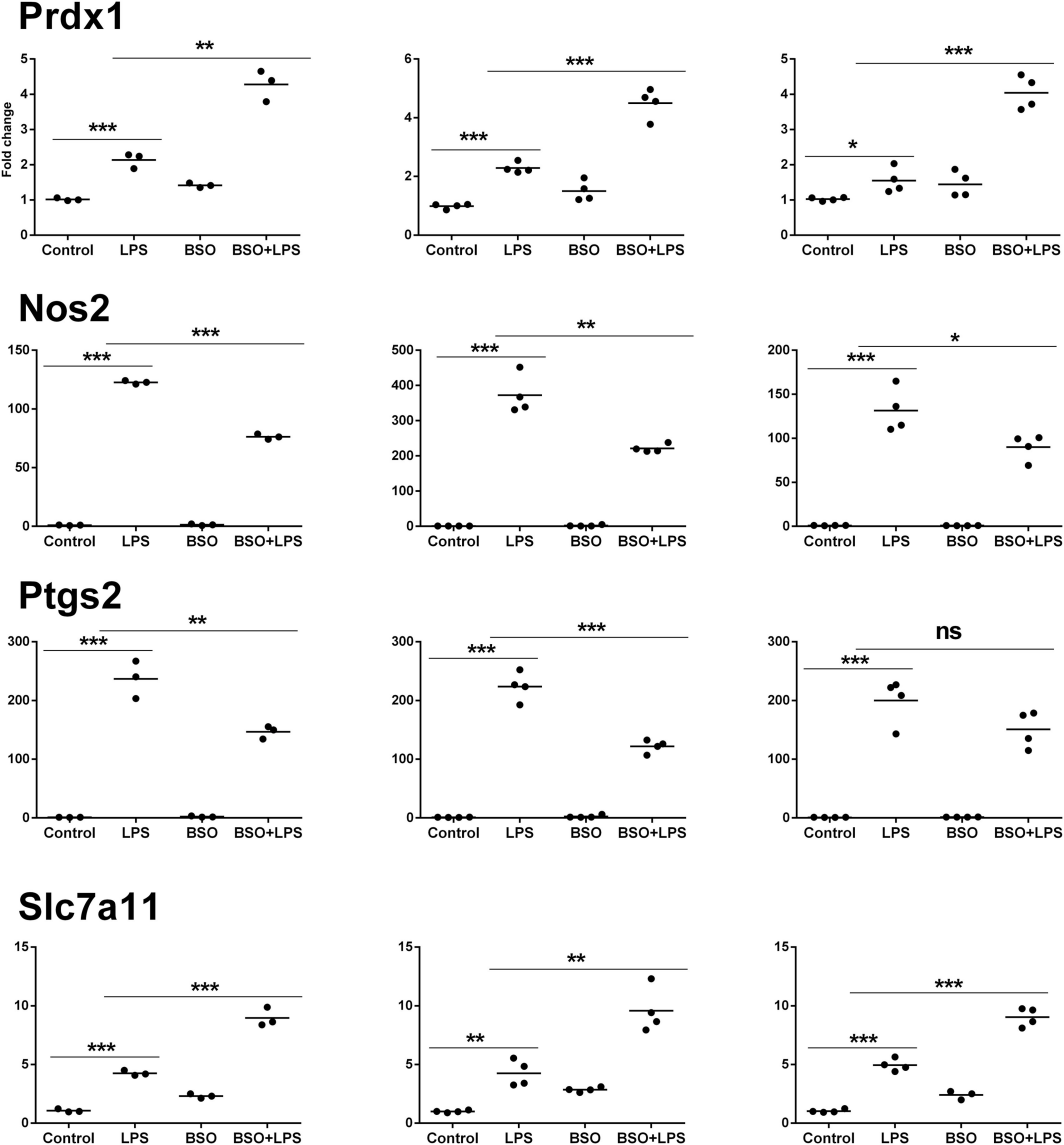
PCR validation of the microarray data at 6 h. Results for four genes (Prdx1, Nos2, Ptgs2, Slc7a11), comparing expression data from microarrays (*N* = 3 biological replicates; left graphs) with results from PCR analysis using all the four replicates of the RNA from the same experiment (*N* = 4 biological replicates; middle graphs) and RNA from an independent experiment (*N* = 4 biological replicates; right graphs). Data are expressed as fold change vs one of the respective control samples. For each experimental group, the mean is also shown. *P < 0.05, **P < 0.01, and ***P < 0.001 by two-tailed Student’s *t*-test. PCR, polymerase chain reaction.

Microarray results were validated by RT-qPCR for 11 genes (Figures 6 and 7). Ten genes were selected from Groups 1 or 2, at 2 and 6 h. In addition, by manually checking the gene list, we noticed that Nos2 was included in Group 2 at 6 h (induced by LPS and inhibited by BSO); however, it passed the first threshold (*P* < 0.05 by Student’s *t*-test, as shown in Figure 7) but not the more stringent statistical analysis using correction for multiple comparisons. Since we have a specific interest in this gene, it was selected for validation by RT-qPCR.

We performed validation in two sets of samples: one with the same RNA used for the microarray experiment (qPCR1) and one with RNA from an entirely independent experiment (qPCR2). For all 11 genes tested, PCR confirmed the differential expression detected by microarray analysis both at 2 and 6 h (Figures 6 and 7, respectively). In the second experiment, at 2 h results were confirmed for five out of seven genes, including il1b, Irf9, Mx2, Il4i1, and Srxn1; at 6 h, three genes out of four were validated including Prdx1, Nos2, and Slc7a11. Interestingly, by RT-qPCR we could find a statistically significant inhibitory effect of BSO on LPS-induced Nos2, which did not pass the correction for multiple comparisons; this is not surprising, since the false discovery rate correction, being more conservative, can generate false negatives. We decided to show the more reliable results obtained in the two independent experiments assayed by PCR (Figure 7); however, for consistency, Nos2 was not included in any subsequent analysis (functional analysis, TF analysis), and is not listed in File S1 in Supplementary Material.

### LPS Induces an Antiviral Response Dependent on GSH

We wondered whether the GSH requirement in the induction of genes in the IFN response pathway in Group 2 was biologically relevant. Therefore, we investigated the effect of LPS on PR8 influenza virus infection in RAW cells in which GSH had been depleted by BSO.

**Figure 8:**
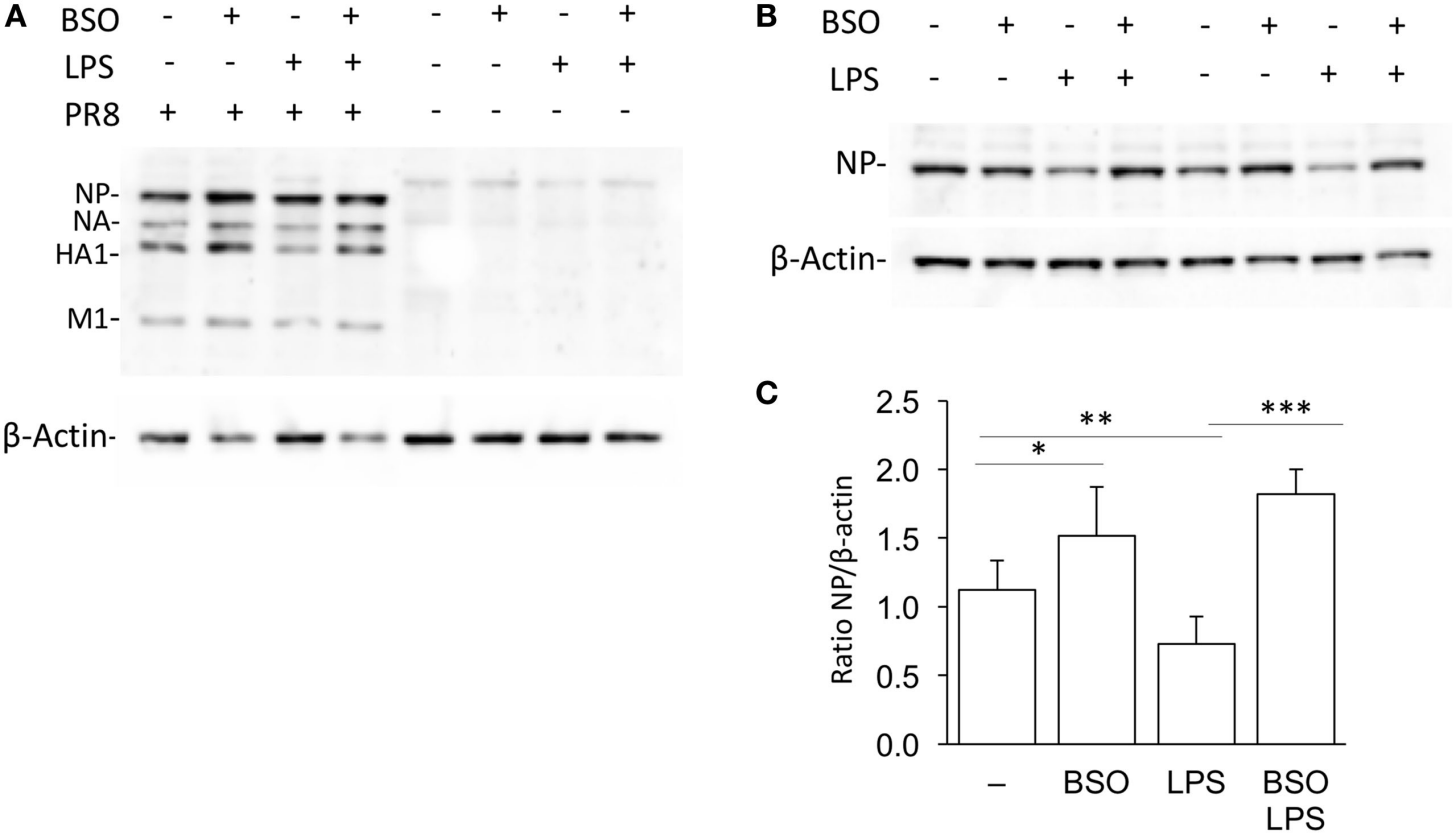
LPS activation of antiviral innate immunity is dependent on GSH. **(A)** Western blot for influenza virus proteins in RAW cells infected with PR8 or uninfected, after LPS treatment, with and without GSH depletion. β-Actin was used as loading control. Representative of two Western blots (*N* = 2 biological replicates). **(B)** Levels of NP viral protein in RAW cells pretreated with LPS, with and without GSH depletion. Representative of four Western blots (*N* = 8 biological replicates from two independent experiments). **(C)** Densitometric analysis expressed as the mean ± SD of the ratio NP/β-Actin from eight biological replicates from two independent experiments for a total of four Western blots. *P < 0.05, **P < 0.01, and ***P < 0.001 by two-tailed Student’s *t*-test. GSH, glutathione; LPS, lipopolysaccharide; NP, nucleoprotein.

As shown in Figure 8, when cells were infected with PR8, LPS reduced infection, in terms of intracellular viral protein production; influenza nucleoprotein (NP, the most expressed among the viral proteins) was significantly decreased in cells pretreated with LPS. However, the effect of LPS was not observed in GSH-depleted cells. Although, as reported previously, BSO alone increased NP production (21), the treatment with both LPS and BSO induced a further significant increase.

### Effect of ROS and Thiol Antioxidants on the Induction of Group 1 and Group 2 Genes by LPS

**Figure 9:**
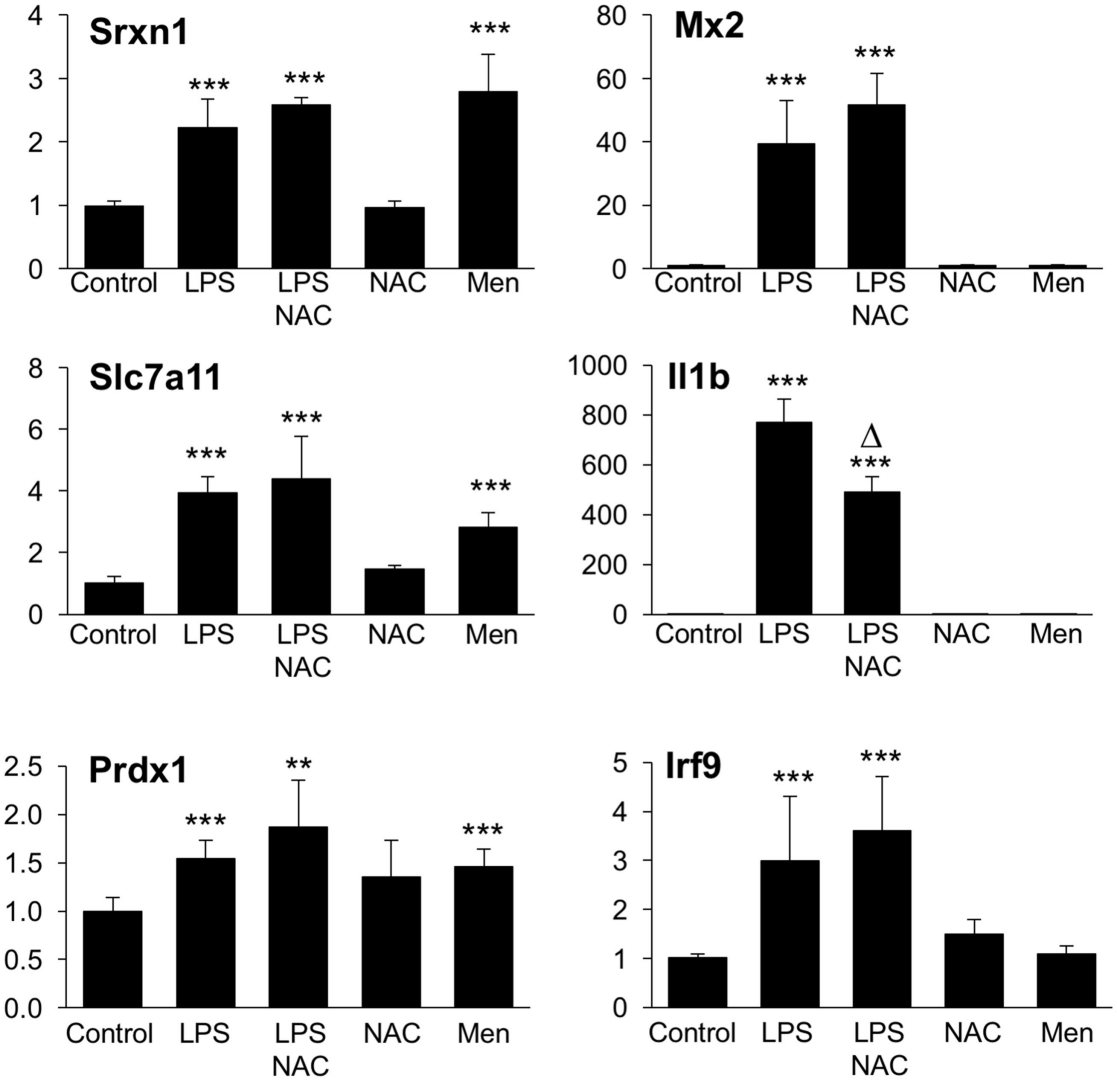
Effect of NAC and menadione on Group 1 (left) and Group 2 (right) genes. Cells were treated with 5 mM NAC for 1 h and then stimulated with 10 ng/ml LPS for further 2 h. Menadione (Men) was added at 10 µM for 2 h. Gene expression was measured by qPCR. Data are expressed as fold change vs one of the control samples, and are the mean ± SD of six biological replicates from two independent experiments. **P < 0.01 and ***P < 0.001 vs control; Δ *P* < 0.01 vs LPS by two-tailed Student’s *t*-test. LPS, lipopolysaccharide; NAC, *N*-acetyl-l-cysteine; qPCR, quantitative polymerase chain reaction.

We next asked the question whether the inhibitory effect of GSH on Group 1 genes, as revealed by the upregulation by BSO, might be due to its ROS-scavenging antioxidant action. To answer this, we first investigated whether the induction of Group 1 genes by LPS was inhibitable by the thiol antioxidant NAC. Second, to investigate whether ROS generation induced by LPS could have a role in the induction of Group 1 genes, we asked whether a ROS-generating agent (menadione) would reproduce the effect of LPS. As shown in Figure 9, NAC did not alter the induction of selected Group 1 genes (Srx1, Prdx1, Slc7a11). On the other hand, all these genes were induced by menadione alone.

The same experimental framework was used to study the relevance of the ROS scavenging properties of GSH in its permissive role for the induction of Group 2 genes. As shown in Figure 9, NAC did not increase further, or affect in any way, the LPS induction of selected Group 2 genes (Mx2 or Irf9); on the contrary, NAC inhibited by about 45% the induction of il1b. Opposite to what observed with Group 1 genes, menadione by itself was unable to regulate the expression of any of Group 2 genes measured.

## Discussion

This study supports the view that endogenous GSH plays a pivotal role for the establishment of the innate immune responses to viruses, possibly acting as a signaling molecule with a mechanism different from simple scavenging of ROS. Overall, a 99% decrease in GSH levels had a minimal impact on the gene expression profile of LPS-treated macrophages; LPS regulated the expression of about 15% of the transcripts, of which only less than 4% (i.e., 0.6% of the total) were affected by BSO (Figure 2). The fact that the vast majority of transcripts were unaffected by BSO is also an indirect confirmation that, within the concentrations and incubation times used, BSO does not have significant toxic or non-specific effects.

Of the genes belonging to the category “inflammatory response” (GO:0006954), comprising several inflammatory cytokines that were induced by LPS in our model (60 transcripts at 2 h and 64 at 6 h), only one gene (CXCL10) was upregulated by BSO. The observation that GSH depletion does not exacerbate the transcription of inflammatory genes, at least in our experimental conditions, might seem at variance with the existing literature starting from pioneering paper by Schreck et al. (24), reporting that ROS activate NF-kB and increase several inflammatory genes, while thiol antioxidants inhibit their expression (25). However, most of that evidence is based on *in vitro* or *in vivo* experiments using exogenously administered thiol antioxidants or pro-oxidants. What our data do not support is the extrapolation of evidence from those experiments to the conclusion that GSH is an endogenous anti-inflammatory molecule through its ROS-scavenging activity. In fact, previous reports noted that exogenous GSH or its precursor NAC inhibits the production and expression of TNF, IL-6, and IL-8 by LPS-stimulated macrophages in the absence of any significant change in intracellular GSH (25). The results reported here are also in agreement with our previous studies where we observed that there are more H_2_O_2_-induced genes that require GSH for their upregulation than genes whose induction by H_2_O2 is exacerbated by GSH depletion (26). Interestingly, in that study using human monocytic cells, many of the H_2_O_2_-induced genes for which GSH had a facilitatory role were related to immunity (26).

In addition, the only LPS-induced transcripts mapping to innate immunity in their functional annotation were inhibited, rather than upregulated, by GSH depletion (Group 2 genes). Not only innate immunity genes in Group 2 require GSH for their induction but also they were not induced by ROS alone (using menadione as a ROS-generating chemical) and their LPS induction was not inhibited by NAC, ruling out the possibility that ROS act as signaling molecules in their induction by LPS. The only exception was il1b whose LPS induction was inhibited by NAC but was also inhibited by GSH depletion, suggesting that GSH is important for IL-1b induction by LPS but possibly not through an antioxidant mechanism because (i) exogenous NAC and endogenous GSH appear to have an opposite role, and (ii) an oxidant alone does not induce IL-1b expression. In line with these findings, it has been shown that molecules altering intracellular thiol content with different mechanisms (i.e., GSH vs NAC derivatives) are able to influence differently LPS-induced pathways (7).

**Figure 10:**
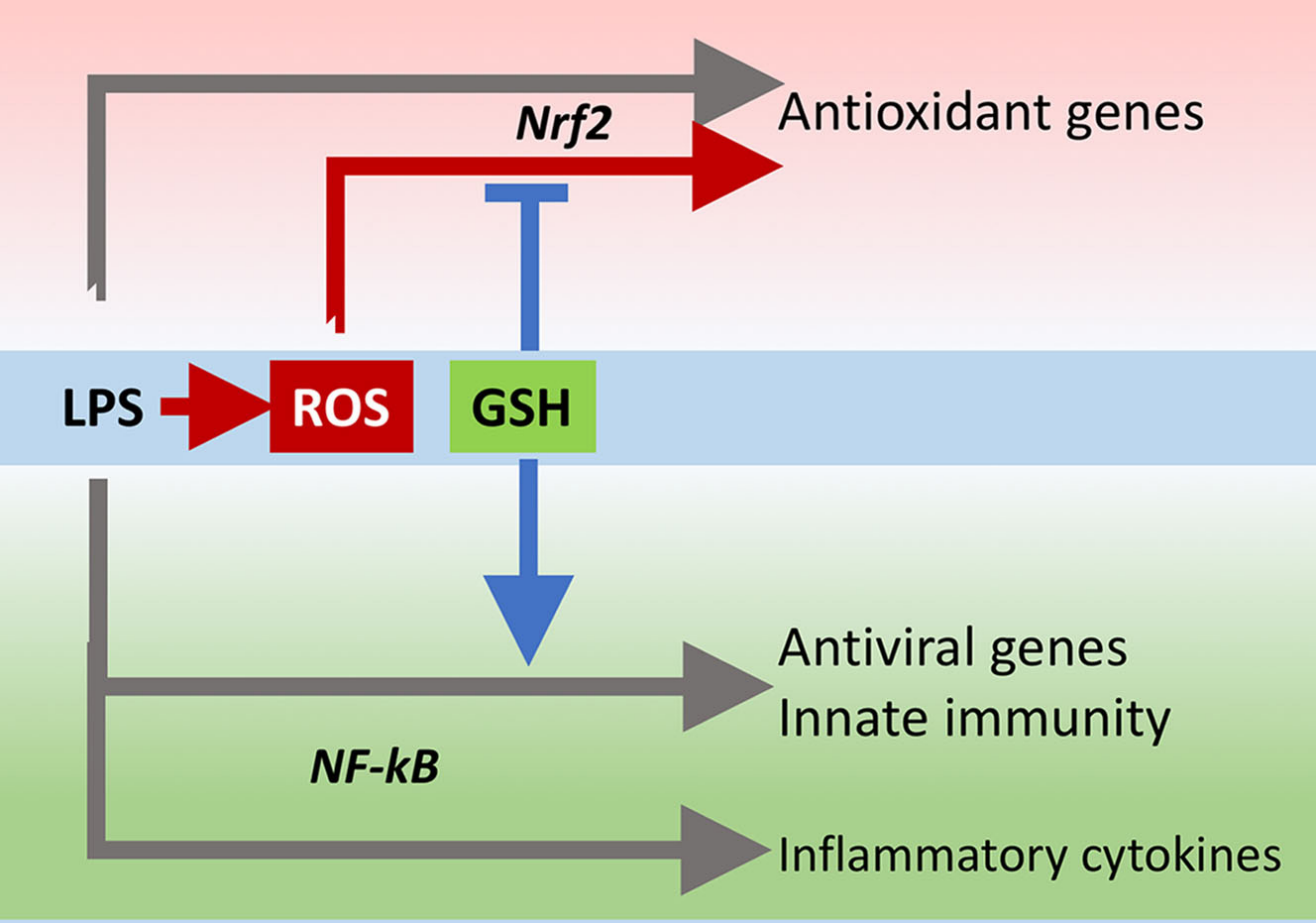
GSH fine-tuning of TLR4 signaling. LPS triggers TLR4 to induce gene expression of inflammatory cytokines, antioxidant genes, and antiviral/immunity pathways. GSH orients the TLR4-mediated changes in gene expression profile toward activation of host defense. GSH, glutathione; LPS, lipopolysaccharide; TLR4, toll-like receptor 4.

The innate immune response is also important for antiviral defense and activation of TLR4 leads to induction of antiviral proteins including IFNs and IFN-related genes (27, 28) such as MxA and Oas (29, 30). Our data, although obtained in a model where infectivity was low, suggest that GSH is important for the activation of an antiviral response. This happens without affecting inflammatory genes, except for IL-1b whose induction was also facilitated by the presence of GSH. There is evidence for a fine-tuning of TLR signaling (31), and these data indicate that GSH may be important in directing it toward specific small patterns of genes implicated in host defense rather than toward those responsible for the inflammatory response, as outlined in Figure 10.

The behavior of genes in Group 1 is what one would expect. They include enzymes for GSH synthesis and antioxidant enzymes such as Prdx1, Srxn1, and Hmox. All these genes map to nrf2, a master regulator of redox homeostasis (32). Their regulation by BSO is in accordance with the hypothesis that endogenous GSH acts as an ROS scavenger because menadione induces their expression. However, NAC did not inhibit their induction by LPS, suggesting that LPS induces nrf2 target gene expression independently of the increase in ROS production. This agrees with a recent study by Cuadrado et al. showing that LPS can activate nrf2 *via* the small GTPase RAC1, independently of ROS (33).

In this picture, endogenous GSH might be important through other mechanisms than just scavenging ROS. In fact, nrf2 activation is dependent on oxidation of its redox sensor, keap1. While keap1 oxidation is mainly studied using ROS or various electrophiles, its thiol groups can also be oxidized by GSSG through a thiol/disulfide exchange reaction (34). It is therefore possible that the change in GSH/GSSG ratio caused by BSO (Figure 1) causes nrf2 activation by oxidation of keap1 and this adds up to the RAC1-dependent activation by LPS.

Several studies have indicated that activation of nrf2 by administration of electrophilic compounds has an anti-inflammatory effect and decreases LPS-induced transcription of other NF-kB target genes, including TNF, IL-1b, and IL-6, in RAW cells (35, 36). However, as mentioned earlier, in our experimental conditions in which nrf2 was likely activated by GSH depletion, as suggested by the increased expression of nrf2 target genes, we have not observed an effect on any inflammatory cytokine other than IL-1b. Once again, the difference might be that we did not use exogenous electrophiles to induce nrf2.

This highlights one point that is often overlooked. GSH is not just an antioxidant that participates in ROS elimination (either *via* its direct ROS scavenging activity or as a substrate for GSH peroxidases) but, like any other thiol including NAC, is also a reducing agent, as well as GSSG is a thiol oxidizing agent. Therefore, these two molecular species, GSH and GSSG, can regulate biological pathways in a redox-dependent manner, independently of ROS scavenging. This could happen by reversible oxidoreduction of protein thiol/disulfides, as described for keap1, but also by formation of mixed disulfides between GSH and protein cysteines. In fact, protein glutathionylation is a major mechanism of redox regulation of immunity (10, 37), affecting the function of key proteins including NF-kB (38), STAT3 (39), PKA (40), TRAF3, and TRAF6 (41), as well as participating in the release of danger signals (42, 43).

On the other hand, redox regulation often implies a role for the production of low levels of “regulatory” ROS (9). However, in this experimental model, the induction of host defense genes in Group 2 (at least those shown in Figure 7, il1b, Mx2, and Irf9) is inhibited by BSO, evidencing the need for GSH, but is not amplified by NAC, suggesting that scavenging LPS-induced ROS is not the main mechanism of action of endogenous GSH.

The finding that several genes that are important for the antiviral response, mostly part of IFN signaling pathways, including the antiviral proteins Oas and Mx2, require GSH for optimal induction by LPS adds knowledge to previous findings, indicating that GSH can inhibit viral infection (44, 45) and that viral infection causes release of glutathionylated thioredoxin and Prdx (46).

There is a large body of evidence showing the importance of GSH in immunity, including antiviral immunity (47), but so far this was ascribed to its action as ROS scavenger to inhibit oxidative stress. The present study indicates that GSH has other important signaling roles independently of protection from oxidative stress, and its action may not be vicariated by another thiol antioxidant. It might even be hypothesized that the “oxidative stress,” and consequent GSH depletion, caused by a virus as a direct consequence of its replication cycle (48) and implicated in the pathogenesis of the disease (49, 50), could be a way by which the virus attempts to diminish the antiviral response by impairing GSH-dependent antiviral pathways.

However, to understand the validity of our conclusions to other models, one needs to bear in mind the limitations of this study that is investigating mRNAs in a cell line. Future studies will need to measure the proteins of interest (for instance, IL-1b) to see whether the changes observed at the level of transcripts are reflected in changes in protein levels. To generalize the relevance of this mechanism, the observation will need to be confirmed in primary cells, including human cells, and possibly *in vivo*.

## Author Contributions

MD, PC, MM, IC, LC, FP, and KA performed experiments. AH, PG, KA, LC, MM, FP, and AP designed and supervised experiments. MD, PG, MM, FP, and PC wrote the paper.

## Conflict of Interest Statement

The authors declare that the research was conducted in the absence of any commercial or financial relationships that could be construed as a potential conflict of interest.

## Footnotes

^1^http://www.ncbi.nlm.nih.gov/geo.

^2^https://david.ncifcrf.gov.

## Supplementary Material

File S1 Full list of the 124 + 177 transcripts from Figure 2. Transcripts most affected by BSO among those significantly regulated by LPS (BSO + LPS vs LPS alone, FC > 1.5, *P* < 0.05 from Figure 2, at both time points). Transcripts in bold are those also significantly affected by BSO alone (BSO vs control, with a cutoff of FC 1.5, *P* < 0.05). The log_2_-transformed gProcessed signals of the three biological replicates are shown. The FC between the two groups indicated is expressed as log_2_ ratio. Statistical significance is calculated by one-way ANOVA followed by a correction for multiple comparisons as indicated in Section “Materials and Methods.” ANOVA, analysis of variance; BSO, buthionine sulfoximine; FC, fold change; LPS, lipopolysaccharide.

File S2 NF-kB target genes upregulated by LPS. For each transcript, the table reports the log_2_-transformed average gProcessed signal from three biological replicates, the fold change between the various experimental groups expressed as log_2_ ratio (LPS vs control and BSO/LPS vs LPS) and the respective *P*-value for each comparison, obtained by two-tailed Student’s *t*-test. Transcripts in bold are those in Group 2 (significantly affected by BSO with a cutoff of 1.5-fold change and *P* < 0.05). BSO, buthionine sulfoximine; LPS, lipopolysaccharide.
