# Supplementary material for: Glutathione Fine-Tunes the Innate Immune Response toward Antiviral Pathways in a Macrophage Cell Line Independently of Its Antioxidant Properties
Source: Front Immunol. 2017 Sep 29;8:1239. doi: 10.3389/fimmu.2017.01239 (PMC5626850; doi:10.3389/fimmu.2017.01239)
Supplement: Supplementary file 3 [file table_1.docx]

| **Supplementary Table 1. LPS-downregulated transcripts most affected by BSO in groups 3 and 4** | | | | | | | | |
| --- | --- | --- | --- | --- | --- | --- | --- | --- |
| **2 h** | | | | **6 h** | | | | |
| **GeneSymbol** | **GenbankAccession** | **P value** | **log_2_FC** |  | **GeneSymbol** | **GenbankAccession** | **P value** | **log_2_FC** |
| **Most up-regulated by BSO (Group 3)** | | | | | | | | |
| Enho | NM_027147 | 0.0005 | 1.81 |  | 4930415C11Rik | AI606402 | 0.0016 | 1.71 |
| Kcnma1 | NM_001253364 | 0.0032 | 1.53 |  | AU019990 | NR_033469 | 0.0001 | 1.70 |
| Sirpb1b * | NM_001173460 | 0.0130 | 1.48 |  | AI661453 | NM_145489 | 0.0079 | 1.64 |
| Tmem116 | AK039773 | 0.0042 | 1.43 |  | Clec4a4 | NM_001005860 | 0.0015 | 1.43 |
| Tmem51 | NM_145402 | 0.0002 | 1.37 |  | Clec4n * | NM_020001 | <0.0001 | 1.34 |
| Rnf150 | NM_177378 | 0.0052 | 1.36 |  | Abhd15 | NM_026185 | 0.0239 | 1.33 |
| AI844869 * | BE862015 | <0.0001 | 1.29 |  | Zfpm2 | NM_011766 | 0.0046 | 1.32 |
| Zfp318 | NM_021346 | 0.0022 | 1.21 |  | D930027P08Rik | AK086428 | 0.0055 | 1.30 |
| Osgin1 * | NM_027950 | <0.0001 | 1.18 |  | Osgin1 | NM_027950 | <0.0001 | 1.26 |
| Bcl2l14 | NM_025778 | 0.0042 | 1.13 |  | 5430425K12Rik * | NR_103550 | 0.0033 | 1.24 |
| Ypel2 | NM_001005341 | 0.0041 | 1.10 |  | Prss46 | NM_183103 | <0.0001 | 1.23 |
| 4930556M19Rik | AK047938 | 0.0088 | 1.09 |  | Arhgap8 | NM_028455 | <0.0001 | 1.20 |
| Rcvrn * | NM_009038 | 0.0021 | 1.03 |  | Zfyve28 | NM_001015039 | <0.0001 | 1.13 |
| AA415398 | NM_001004178 | 0.0006 | 0.95 |  | Rhox2h | NM_001100465 | 0.0112 | 1.09 |
| P4ha2 | NM_011031 | 0.0090 | 0.95 |  | Pcdhb6 | NM_053131 | 0.0005 | 1.08 |
| **Most down-regulated by BSO (Group 4)** | | | | | | | | |
| Gm10778 | NM_001142963 | 0.0107 | -1.04 |  | Maf | NM_001025577 | 0.0006 | -1.33 |
| 1600014C10Rik | NM_028166 | 0.0002 | -1.01 |  | Sulf2 | NM_028072 | 0.0009 | -1.29 |
| Gm9779 | BC006743 | 0.0213 | -0.85 |  | C1qa * | NM_007572 | 0.0007 | -1.24 |
| Slc25a40 | XR_376693 | 0.0059 | -0.80 |  | C1qc * | NM_007574 | <0.0001 | -1.09 |
| C1qa * | NM_007572 | 0.0043 | -0.75 |  | Slc9a3r2 | NM_023055 | 0.0059 | -0.92 |
| 1700027A07Rik | AK006404 | 0.0068 | -0.72 |  | Pi16 | NM_023734 | 0.0005 | -0.92 |
| A430106G13Rik | AK053260 | 0.0025 | -0.64 |  | Cx3cr1 * | NM_009987 | 0.0002 | -0.82 |
| Themis2 | NM_001033308 | 0.0023 | -0.61 |  | Cx3cr1 * | NM_009987 | 0.0002 | -0.81 |
|  |  |  |  |  | Cx3cr1 * | NM_009987 | 0.0037 | -0.69 |
|  |  |  |  |  | Sepp1 | NM_001042614 | 0.0044 | -0.69 |
|  |  |  |  |  | Fgf11 | XM_006532188 | 0.0002 | -0.67 |
|  |  |  |  |  | Cenpf | NM_001081363 | 0.0016 | -0.67 |
|  |  |  |  |  | Frat2 | NM_177603 | <0.0001 | -0.64 |
|  |  |  |  |  | 4831440E17Rik | NR_030700 | <0.0001 | -0.62 |
|  |  |  |  |  | Tob1 | NM_009427 | 0.0024 | -0.59 |

List of the 15 LPS-decreased transcripts most affected by BSO (BSO+LPS vs LPS alone, FC>1.5, P<0.05 by one-way ANOVA, followed by a correction for multiple comparisons as indicated in the Methods section) in groups 3 and 4 from Figure 2, at both time points. * Transcripts that are also significantly affected by BSO alone. The full list can be seen in Supplementary File 1.
